# High Thermal Conductivity Diamond–Copper Composites Prepared via Hot Pressing with Tungsten–Coated Interfacial Layer Optimization

**DOI:** 10.3390/ma18163882

**Published:** 2025-08-19

**Authors:** Qiang Wang, Zhijie Ye, Lei Liu, Jie Bai, Yuning Zhao, Qiang Hu, Hong Liu, Lang Hu, Xiaodong Guo, Yongneng Xiao, Wenxin Cao, Zhenhuai Yang

**Affiliations:** 1 School of Aeronautics, Chongqing Jiaotong University, Chongqing 400074, China; 2 Key Laboratory of Low-Grade Energy Utilization Technologies and Systems, Chongqing University, Ministry of Education, Chongqing 400044, China; 3 Zhengzhou Research Institute, Harbin Institute of Technology, Zhengzhou 450000, China; 4 Laboratory of Microwave and Vacuum Technology, Jihua Laboratory, Foshan 528200, China

**Keywords:** diamond–copper composites, thermal conductivity, interfacial thermal conductance, hot-pressing

## Abstract

Diamond–copper composites, due to their exceptional thermal conductivity, hold significant potential in the field of electronic device thermal management. Hot-press sintering is a promising fabrication technique with industrial application prospects; however, the thermal conductivity of composites prepared by this method has yet to reach optimal levels. In this study, tungsten was deposited on the surface of diamond particles by magnetron sputtering as an interfacial transition layer, and hot-press sintering was employed to fabricate the composites. The findings reveal that with prolonged annealing time, tungsten gradually transformed into W_2_C and WC, significantly enhancing interfacial bonding strength. When the diamond volume content was 50% and the interfacial coating consisted of 2 wt.% W, 92 wt.% WC, and 6 wt.% W_2_C, the composite exhibited a thermal conductivity of 640 W/(m·K), the highest value reported among hot-press sintered composites with diamond content below 50%. Additionally, the AMM (Acoustic Mismatch Model) and DMM (Diffusion Mismatch Model) models were utilized to calculate the interfacial thermal conductance between different phases, identifying the optimal interfacial structure as diamond/W_2_C/WC/W_2_C/Cu. This composite material shows potential for application in high-power electronic device cooling, thermal management systems, and thermoelectric conversion, providing a more efficient thermal dissipation solution for related devices.

## 1. Introduction

With the advent of the intelligent era, the use of electronic devices has increased exponentially in recent years, particularly within high-tech fields such as aerospace and mobile chip manufacturing. As electronic components continue to achieve higher integration and power density, traditional thermal management materials have become inadequate for meeting these devices’ heat dissipation requirements [1]. Diamond, possessing a thermal conductivity of up to 2000 W/(m·K), holds substantial promise for enhancing heat dissipation in electronic devices. However, due to its high hardness and processing challenges, the prevailing approach involves combining copper (with a thermal conductivity of 400 W/(m·K)) with diamond particles. Theoretically, diamond–copper composites should exhibit high thermal conductivity. Yet, the diamond particles and copper matrix display poor chemical compatibility and wettability at their interface; even at 1100 °C, the contact angle remains high at approximately 130°, often leading to non-bonding [2]. Such composites with only mechanical bonding typically suffer from weak interfacial adhesion. This creates defects at the interface, consequently degrading the thermal conductivity of the composite material. Therefore, resolving the interface bonding problem between copper and diamond, as well as clarifying the structure–activity relationship that governs the thermal physical properties of the connection interface, is of great significance for the development of high-performance diamond–copper composite materials.

Recent studies identify two primary approaches to enhance the thermal conductivity of diamond–copper composites: diamond surface metallization and matrix alloying. Both methods aim to create a carbide transition layer between the copper and diamond, thereby improving phonon energy transfer efficiency across this interface [3]. Researchers have tested various transition layers—including B [4,5], Si [6], Cr [7,8,9], Ti [10,11], Mo [12], and W [13,14,15]—to optimize composite thermal conductivity. While both metallization and alloying have improved thermal conductivity to some extent, optimizing the transition layer composition remains challenging. Different materials significantly impact performance; tungsten (W), for instance, is a key focus due to its high melting point and excellent thermal stability. Thermal diffusion-produced composites achieved 559 W/(m·K) [16], hot-pressed ones reached 661 W/(m·K) [15], and vacuum-infiltrated composites attained 874 W/(m·K) [17]. Tungsten carbide exhibits the highest intrinsic thermal conductivity among carbides, has negligible solubility in copper, and forms a wetting angle with copper of approximately 17°, significantly lower than other carbides, making it an excellent interfacial layer choice [18]. Additionally, tungsten forms stable carbides with diamond at high temperatures, enhancing interfacial bonding strength and potentially improving thermal conductivity by modulating phonon scattering [19]. However, the formation mechanism of tungsten carbides at the interface of diamond–copper composites, as well as the structure and distribution of the generated tungsten carbides, remain to be further elucidated. This will facilitate the understanding of the principle by which the tungsten carbide transition layer at the interface enhances the thermal performance of the composite materials.

Previous studies demonstrate that researchers have successfully fabricated diamond–copper composites with thermal conductivities exceeding 800 W/(m·K) using spark plasma sintering (SPS) [20,21] and gas pressure infiltration (GPI) [22], confirming these methods’ effectiveness for enhancing thermal conductivity. By contrast, hot-press sintering (HPS) [23,24,25] achieves uniform microstructures and dense interfacial bonding through precise temperature and pressure control. This significantly reduces porosity, thereby improving overall composite performance. HPS is also compatible with diamond particles of diverse shapes and sizes, offers simpler processing, lower equipment costs, and operates under vacuum or inert atmospheres, effectively preventing copper oxidation. Consequently, HPS not only ensures high thermal conductivity but also offers distinct advantages in process control, fabrication uniformity, and interfacial bonding quality, making it highly promising for engineering applications.

In this work, the surface of diamond powder was treated by combining magnetron sputtering and annealing. Subsequently, a copper layer was formed on the surface through chemical deposition. Finally, a diamond–copper composite material with excellent thermal conductivity was prepared using HPS. The relationship between the thermal physical properties of the diamond–copper composites and the tungsten–carbonitride structures and distribution at the interface was characterized and analyzed. Through the application of AMM and DMM, the interfacial thermal conductivity of the composites was theoretically discussed, and the optimal interface structure and thermal conduction mechanism were further clarified. This provided a theoretical basis for optimizing the performance of the diamond–copper composite materials.

## 2. Experiment

### 2.1. Sample Preparation

**Figure 1:**
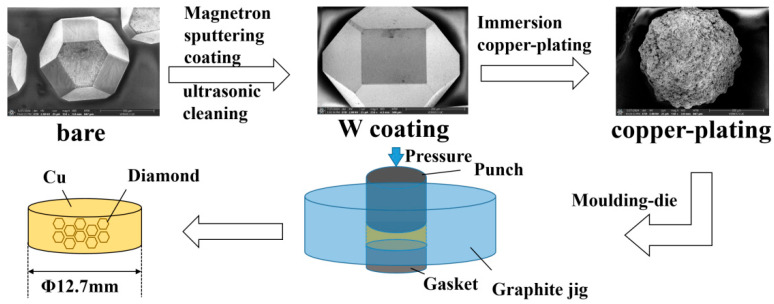
Flowchart of diamond–copper composite preparation.

The sample preparation process is shown in Figure 1. Diamond particles with an average diameter of 400 μm (HHD-90, Huanghe Whirlwind Co., Ltd., Xuchang, China) were used as the substrate for coating deposition. Initially, the diamond particles were thoroughly cleaned by sequentially immersing them in solutions of nitric acid, sodium hydroxide, acetone, and anhydrous ethanol. Ultrasonic cleaning was applied to remove surface oils and impurities, after which the particles were dried in an oven for later use.

The diamond particles were then placed in a magnetron sputtering device (PVD8090, Lebuy Vacuum Technology Co., Ltd., Shenyang, China) and subjected to pulsed magnetron sputtering in an argon atmosphere, depositing a tungsten coating with an average thickness of 200 nm on the particle surfaces. To ensure complete coating, the particles were placed on a tilted rotating plate to achieve uniform coverage.

After metallization, the particles were annealed at 1100 °C for varying durations until all W layers had fully converted to tungsten carbide (WC). To prevent oxidation during annealing, the vacuum pressure in the tube furnace (SK-G15123K-2-610, Zhonghuan Electric Furnace Co., Ltd., Tianjin, China) was maintained at 5 × 10^−4^ Pa, with a heating rate of 10 °C/min. Subsequently, the annealed diamond particles were activated and sensitized, then placed in a rotary evaporator where a copper coating was chemically deposited on the metallized diamond surfaces. Finally, hot-press sintering was used to form a diamond–copper composite material with a diameter of 30 mm and a thickness of 1.5 mm. The composite was laser cut into 12.7 mm diameter discs to ensure uniform properties throughout the material. The diamond volume fraction in the composite was approximately 50% (ignoring the coating volume).

### 2.2. Sample Characterization

X-ray diffraction (XRD, Bruker D8 Advance, Karlsruhe, Germany) was used for phase identification, with a 2θ angle range of 20–90°. Quantitative analysis of phase content was performed based on the full width at half maximum (FWHM). The surface morphology of diamond after annealing on the (100) and (111) planes was analyzed using scanning electron microscopy (SEM, Thermo Fisher Verios 5 UC, Waltham, MA, USA). The Raman spectra of the annealed diamond particles were collected using a confocal laser Raman spectrometer (RENISHAW, inVia Qontor, Wotton-under-Edge, UK) with a scattering light source of λr = 532 nm, covering a range of 100–3200 cm^−1^, to observe the carbonization of diamond and tungsten. The thermal conductivity of the diamond particles was estimated based on the concentration of nitrogen impurities [26], with the nitrogen concentration obtained from FTIR absorption spectra collected in the range of 400–4000 cm^−1^, at a resolution of 4 cm^−1^, and with 20 scans. The diamond particles were mixed with KBr, compressed into pellets, and compared with pure KBr samples. The thermal diffusivity of the samples with a diameter of 12.7 mm and a thickness of 1.5 mm was measured at room temperature using the LFA467 (NETZSCH) HyperFlash method, following the principles and methods referenced in [27]. The volume density of the composite materials was measured using a method based on Archimedes’ principle and compared with theoretical density. The thermal conductivity was calculated as the product of density, thermal diffusivity, and specific heat [28]. Finally, high-performance focused ion beam dual-beam scanning electron microscopy (Thermo Fisher Helios 5 UX, Waltham, MA, USA) was used to prepare transmission samples, which were then observed under a high-resolution field emission transmission electron microscope (JEM-F200, Japan Electronics Corporation, Akishima, Tokyo, Japan) to examine the morphology of the interfaces.

## 3. Results and Discussion

### 3.1. Diamond Characterization

**Figure 2:**
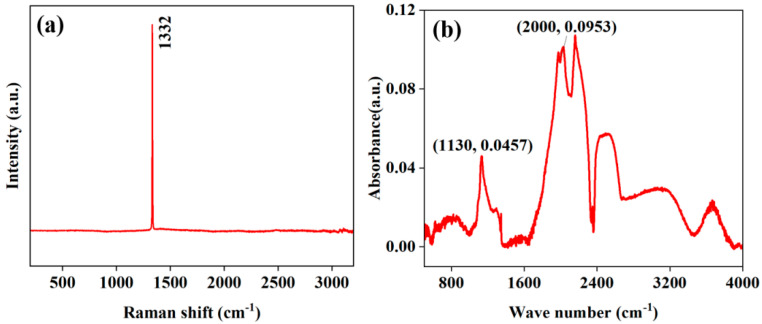
(**a**) Raman spectrum; (**b**) FTIR spectrum of the diamond.

The chemical state of single-crystal diamond particles was investigated using Raman spectroscopy. As shown in Figure 2a, a characteristic peak of the Raman spectrum was detected at 1332 cm^−1^, corresponding to the sp^3^ structure [29]. Nitrogen, as the most common impurity in diamonds (both natural and synthetic), directly affects the physicochemical properties of diamonds. The nitrogen content [N] in diamonds has a quantitative relationship with the thermal conductivity *λ* [26,30]:

(1) λ=2200−3.27N

The nitrogen content in diamonds was calculated using FTIR spectroscopy:

(2) $$N=308\frac{A_{1130}}{A_{2000}}$$

where $A_{1130}$ and $A_{2000}$ represent the absorption intensities at 1130 cm^−1^ and 2000 cm^−1^, respectively. Based on the FTIR spectrum in Figure 2b, the nitrogen content was calculated to be 147 ppm. Substituting the calculated nitrogen content into Equation (1), the thermal conductivity of the single-crystal diamond particles was found to be 1717 W/(m·K).

### 3.2. Influence of Annealing on Film Composition

**Figure 3:**
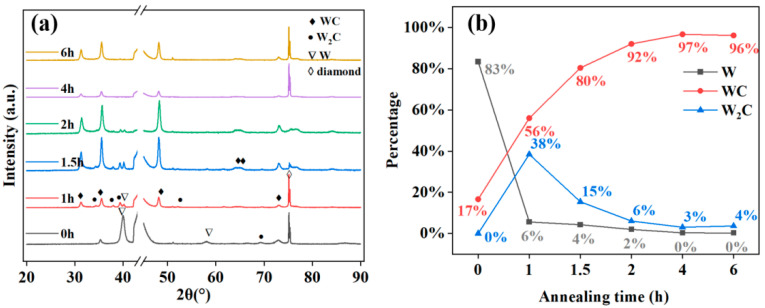
(**a**) XRD patterns of tungsten-coated diamond particles annealed in vacuum at 1100 °C for durations of 0 to 6 h. (**b**) Composition percentage of various phases in the tungsten coating after annealing for 0 to 6 h.

Figure 3a shows the XRD patterns of tungsten-coated diamond particles annealed at 1373 K for durations ranging from 0 to 6 h, obtained using a single light source with a Ni filter. The XRD measurement database includes W (PDF#04-001-0110), W_2_C (PDF#04-003-5411), WC (PDF#04-001-2995), and diamond (PDF#04-008-8218). The composition percentages of the coated W and its carbides were calculated using the K-value method [31], as shown in Figure 3b. The initial sample contained 83% W and 17% WC, which is attributed to the increase in temperature during the sputtering process, where the energy provided by the equipment caused a small amount of W to react with the surface of the diamond, resulting in the formation of WC. During the annealing process, external energy continuously facilitates the dissolution of dislocations and rearrangement of the lattice [32]. After 1 h of annealing, W transformed into the metastable structure W_2_C and the stable WC. As the annealing time increased, the metastable W_2_C gradually converted towards WC, while W also transformed into W_2_C and WC. At 4 h of annealing, W was completely carbonized into W_2_C and WC. However, upon further extending the annealing time, it was observed that W_2_C could not completely convert into WC, resulting in a small amount of W_2_C remaining.

**Figure 4:**
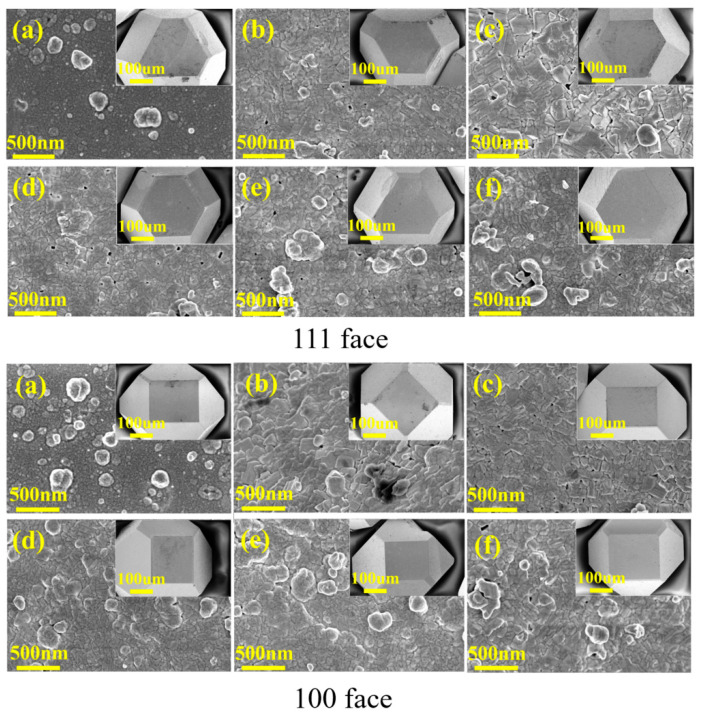
Surface morphologies of tungsten-coated diamond particles annealed at 1100 °C for (**a**) 0 h, (**b**) 1 h, (**c**) 1.5 h, (**d**) 2 h, (**e**) 4 h, and (**f**) 6 h.

As the annealing time of the samples increased, we observed that both the low-surface-energy (111) face and the high-surface-energy (100) face exhibited significant phase transformation of the initially dense metallic tungsten layer into crystalline tungsten carbide (WC) and tungsten dich carbide (W_2_C) under high-temperature annealing, as shown in Figure 4a–f. During this process, the surface morphology transitioned from a continuous smooth structure to a porous structure with distinct grain boundary features (0–1.5 h), with grain sizes gradually increasing. With extended annealing time, the grains began to refine, and the porosity gradually decreased, forming a dense layer of tungsten carbide (1.5–6 h). A longitudinal comparison in Figure 4 reveals that for the same annealing duration, the clustering phenomenon of the particles on the (100) face is more pronounced. As the annealing time increases, the grain growth on the (100) face occurs more rapidly; after 1 h of annealing on the (100) face, the result is comparable to that of 1.5 h on the (111) face, with the (100) face exhibiting fewer pores compared to the (111) face. This difference arises because the surface energy of the (100) face is higher than that of the (111) face. Materials with higher surface energy typically exhibit better wettability and adhesion [33], meaning they can bond more easily with copper. The increase in the grain size of the carbide coating reduces the thermal transfer interface, which promotes the enhancement of thermal conductivity.

**Figure 5:**
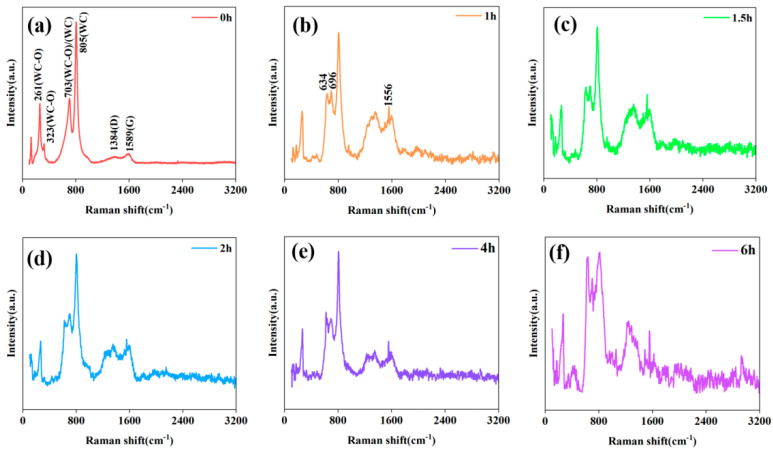
Raman spectra of tungsten-coated diamond samples annealed at 1100 °C for (**a**) 0 h, (**b**) 1 h, (**c**) 1.5 h, (**d**) 2 h, (**e**) 4 h, and (**f**) 6 h.

Figure 5a–f displays the Raman spectra of modified diamond powder samples at different annealing times at 1100 °C, and the identification is performed based on the literature [26,34], as shown in the Table 1. Initially, in the sample (Figure 5a), only the D and G peaks of carbon and the WC phase were observed. Tungsten (W), being a metal, does not exhibit Raman activity. The main peak at 805 cm^−1^ corresponds to the stretching mode of WC, while the three peaks at 261, 323, and 703 cm^−1^ belong to the initial oxidation states of WC (denoted as WC-O). The peak at 703 cm^−1^ also corresponds to the stretching mode of WC.

After annealing, the peak at 703 cm^−1^ (WC-O/WC) splits, forming two peaks at 634 cm^−1^ and 696 cm^−1^. According to the literature [35], this phenomenon is attributed to the further oxidation of WC, as shown in Figure 5b–d. At this point, the thermal conductivity of the composite material begins to increase. Interestingly, the intensities of the 634 cm^−1^ and 696 cm^−1^ peaks change relatively with the extension of annealing time. Before 2 h of annealing, the intensity of the 634 cm^−1^ peak is lower than that of the 696 cm^−1^ peak. However, when the annealing time exceeds 2 h, as shown in Figure 5e,f, the situation is reversed. After 2 h of annealing, the intensity at 634 cm^−1^ begins to exceed that at 696 cm^−1^, indicating that the internal structure of the material further evolves with prolonged annealing time. Although it is currently impossible to quantitatively infer the relationship between the peak intensity ratio and thermal conductivity, the trends observed suggest possible adjustments in the microstructure, which may influence the thermal conductivity of the composite material. Particularly, when the intensity at 634 cm^−1^ is lower than that at 696 cm^−1^, extending the annealing time may facilitate further optimization of the interface, potentially enhancing the thermal performance of the material. This hypothesis requires further experimental validation to more clearly elucidate the relationship between peak intensity changes and improvements in thermal conductivity.

Coincidentally, we observed a significant sharpening and increase in the signals of the D and G peaks after annealing, which is attributed to the reaction between diamond and W at high temperatures. Additionally, we found a particularly sharp peak at 1556 cm^−1^, which the literature [26] suggests could be the g line of graphite, though it is more likely to be a characteristic peak around 1540 cm^−1^. This peak has been observed in diamonds subjected to high-energy ion implantation or deformation under high mechanical stress (in a “diamond” or “bridged graphite” structure). We interpret this as a transitional state of sp^3^-to-sp^2^ bond conversion. Before annealing, the intensity of the D peak was lower than that of the G peak; however, as annealing time increased, the material’s internal structure changed due to the effects of high temperature, resulting in the D peak exceeding the G peak in intensity. As previously mentioned, the carbon on the diamond surface reacts with tungsten, leading to the conversion of sp^3^ bonds to sp^2^ bonds, forming amorphous carbon (1200–1500 cm^−1^), diamond-like distorted graphite (D/G, 1556 cm^−1^), and turbostratic (nanocrystalline) carbon (1384 cm^−1^). According to the literature [34], W_2_C is Raman-inactive.

### 3.3. Thermal Conductivity Performance of Composite Materials

**Figure 6:**
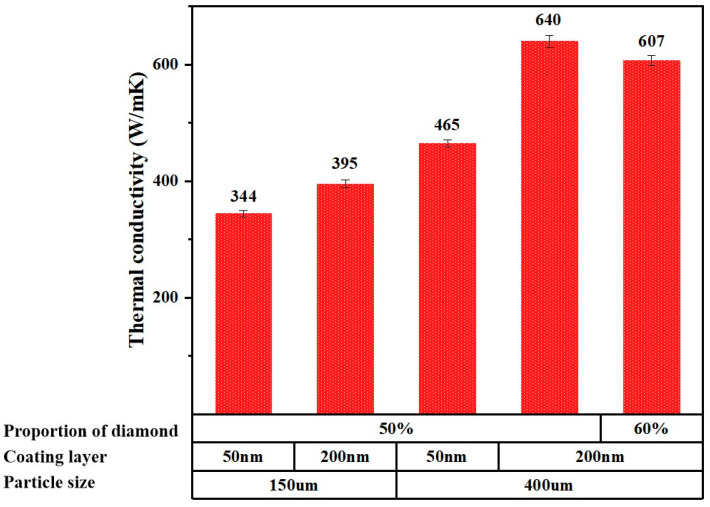
Thermal conductivity of diamond–copper composite materials prepared under different conditions after annealing for 2 h.

Before investigating the effect of coating composition on thermal conductivity, we first conducted a preliminary analysis of the size of diamond particles, coating thickness, and diamond content proportion, as shown in Figure 6. As the particle size increased from 150 µm to 400 µm, the thermal conductivity of the composite material significantly improved. With a coating thickness of 50 nm, the thermal conductivity of 150 µm particles was 344 W/(m·K), while that of 400 µm particles increased to 465 W/(m·K), likely due to the larger particles providing a better thermal conduction pathway. However, under the conditions of 400 µm particles, the thermal conductivity of the 200 nm coating exhibited a wide range of variation (465 W/(m·K) to 640 W/(m·K)), indicating a complex relationship between coating thickness and thermal conductivity. For 150 µm particles, as the coating thickness increased to 200 nm, the thermal conductivity rose from 344 W/(m·K) to 395 W/(m·K); however, under the conditions of 400 µm particles, an increase in coating thickness led to unstable changes in thermal conductivity, with some samples showing a significant decrease, which may be related to the increase in interfacial thermal resistance. Furthermore, the effect of diamond content was not linear: at a 50% content, the thermal conductivity ranged from 344 W/(m·K) to 640 W/(m·K), but when the content increased to 60%, the thermal conductivity dropped to 607 W/(m·K), possibly due to poor contact between particles leading to increased interfacial thermal resistance.

**Figure 7:**
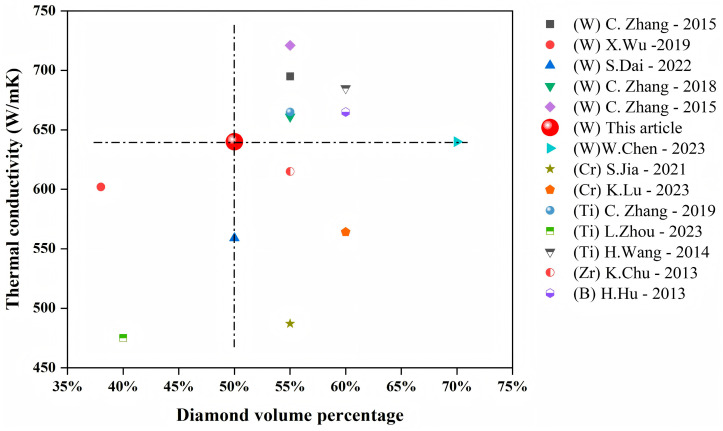
Comparison of diamond volume fraction and thermal conductivity of diamond–copper composite materials prepared by hot pressing sintering method [15,16,23,24,25,38,39,40,41,42,43,44,45].

To validate the thermal conductivity of our diamond–copper composites, we benchmarked our results against the literature data (Figure 7), which presents thermal conductivity values for hot-press-sintered (HPS) composites at varying diamond volume fractions. W coatings achieved 721 W/(m·K) at 55 vol% diamond; Ti coatings reached a peak of 685 W/(m·K); Cr coatings attained 593 W/(m·K); while B and Zr coatings yielded maxima of 665 W/(m·K) and 615 W/(m·K), respectively. Our composite achieved 640 W/(m·K). Although some of the literature samples exceeded this value at >50 vol% diamond, our material containing ≤50 vol% diamond demonstrated superior thermal performance relative to comparative studies. This performance aligns with composites fabricated via pressure infiltration [36] and spark plasma sintering [37] methods. Furthermore, it is particularly important to note that when the content of diamond powder continues to increase, for example, exceeding 50 vol% in this study, there is a risk of cracks appearing in the diamond–copper composite samples after hot-press sintering, and this also undermines the stability of the composites. Collectively, these results confirm that when the HPS method is combined with a reasonable interface design, it can significantly improve the thermal conductivity. This provides a path for optimizing the interface of diamond–copper composites and regulating their high thermal conductivity.

### 3.4. The Effect of Interface Structure on the Thermal Conductivity of Composites

By comparing our data with other studies, we found that the interface bonding has a significant impact on the thermal conductivity of the composites. Therefore, further research on interface characteristics is particularly necessary. Under the conditions of a particle size of 400 μm, a coating thickness of 200 nm, and a diamond volume content of 50%, we investigated the effects of different coating compositions and interfaces on the thermal conductivity of the composites.

The parameters of the diamond–copper composites obtained after HPS are shown in Table 2. To clarify the influence of the interface coating on thermal conductivity, calculations were performed using the H-J model (Hasselman-Johnson model) [12] expressed in Equation (3) and the DEM model (Differential Effective Medium model) expressed in equations [46] (Differential Effective Medium (4) and (5)).

**Table 2:** Thermal diffusivity, specific heat capacity, and density of diamond–copper composites at different annealing times.

Annealing Time (h)	Thermal Diffusivity α (mm^2^/s)	Specific Heat Cp (J/g/K)	Density ρ (g/cm^3^)
0	200.658	0.427	6.38
1	221.844	0.416	6.33
1.5	238.083	0.435	6.15
2	227.891	0.428	6.57
4	207.635	0.401	6.46
6	192.764	0.424	6.41

(3) $$\frac{\lambda_{c}}{\lambda_{m}}=\frac{2(1-V_{d})\lambda_{m}+(1+2V_{d})\lambda_{d}^{eff}}{(2+V_{d})\lambda_{m}+(1-V_{d})\lambda_{d}^{eff}}$$

(4) $$(1-V_{d}){\left(\frac{\lambda_{c}}{\lambda_{m}}\right)}^{1/3}=\frac{\lambda_{d}^{eff}-\lambda_{c}}{\lambda_{d}^{eff}-\lambda_{m}}$$

(5) $$\lambda_{d}^{eff}=\frac{\lambda_{d}}{1+\lambda_{d}/a\cdot h_{c}}$$

In the equations, $\lambda_{c}$ and $\lambda_{m}$ represent the thermal conductivity of the composite material and the matrix, respectively, $V_{d}$ is the volume fraction of the reinforcement, $\lambda_{d}^{eff}$ is the effective thermal conductivity of the reinforcement, and a and $h_{c}$ denote the radius of the reinforcement and the interface thermal conductivity between the reinforcement and the matrix, respectively. $\lambda_{d}$ is the intrinsic thermal conductivity of the reinforcement.

**Figure 8:**
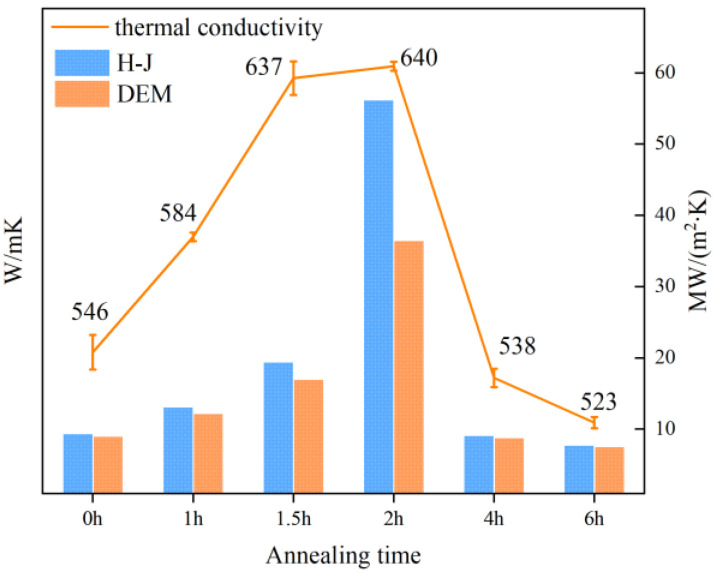
Thermal conductivity of diamond–copper composites prepared at different annealing times, along with the interface thermal conductivity calculated using the H-J model and DEM model.

The measurement and calculation results are shown in Figure 8. As the annealing time increases, the thermal conductivity of the composite first increases and then decreases. The sample obtained through hot-press sintering at 2 h of annealing exhibits the highest thermal conductivity, reaching 640 W/(m·K). Similarly, the interfacial thermal conductivity of the coating is also at its peak. Notably, although the interfacial thermal conductivity of the sample at 1.5 h of annealing is significantly lower than that of the sample at 2 h, its inherently high thermal diffusivity results in a final thermal conductivity that is comparable to that of the sample with the highest thermal conductivity. After the annealing time exceeds 2 h, carbon elements in the diamond begin to graphite, and the sp^3^ bonds convert to sp^2^ bonds, forming amorphous carbon that significantly reduces the interfacial thermal conductivity. Using the H-J model and DEM model for calculations and mutual validation, the interfacial thermal conductivity of the composite increases from 9 MW/(m^2^·K) to 19 MW/(m^2^·K). At 2 h of annealing, the interfacial thermal conductivity of the composite sharply rises to between 36 and 56 MW/(m^2^·K), before subsequently decreasing to around 7 MW/(m^2^·K). To further investigate the influence of the coating interface on the thermal conductivity of the composite, we conducted an in-depth characterization and analysis of the interfacial morphology.

**Figure 9:**
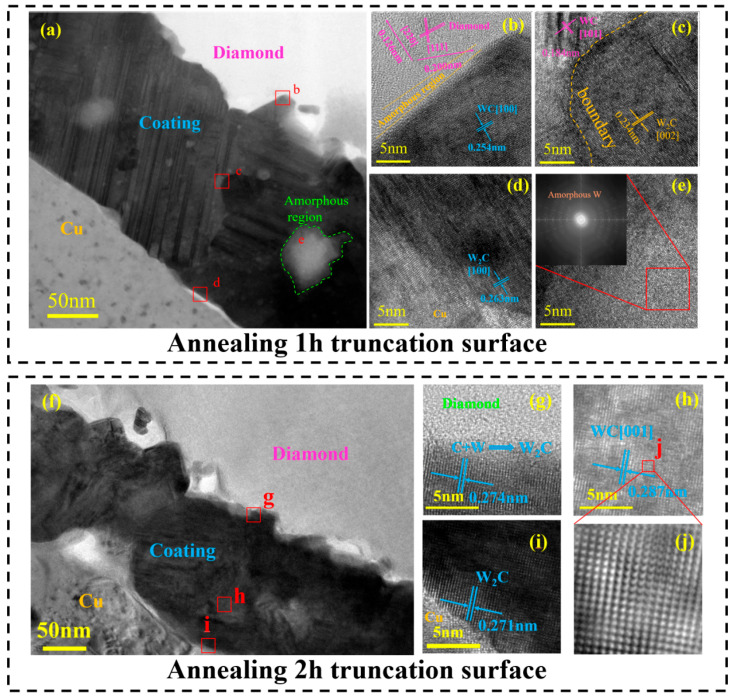
Cross-sectional morphology of samples after 1 h and 2 h of annealing: (**a**) cross-section of the composite material after 1 h of annealing; (**b**) morphology of the diamond–coating interface; (**c**) morphology of the WC/W_2_C interface; (**d**) morphology of the coating–Cu interface; (**e**) morphology of amorphous carbon and its Fourier transform image; (**f**) cross-section of the composite material after 2 h of annealing; (**g**) morphology of the diamond–coating interface; (**h**) morphology of the intermediate coating interface; (**i**) morphology of the coating–Cu interface; (**j**) inverse Fourier transform image of the intermediate coating.

To analyze the influence of interfacial morphology on thermal conductivity in detail, transmission electron microscopy (TEM) was employed for characterization. Figure 9 shows a sample cut from the diamond–copper composite material formed through hot-press sintering after 1 h of annealing using FIB. Figure 9a includes a cross-sectional view of the diamond, coating, and Cu matrix, where good bonding is observed at all interfaces, with no obvious porosity or cracks. Figure 9b–d shows high-resolution images of the interface between the diamond and the coating, the boundary between the coatings of WC/W_2_C, and the coating/Cu interface, respectively. Figure 9e illustrates the amorphous regions formed during the annealing process. Figure 9f also includes the diamond, coating, and copper matrix. Figure 9g–i presents high-resolution images of three regions from Figure 9f. Figure 9j is the inverse Fourier transform image of a region in Figure 9h.

Comparison of the two samples annealed for different durations reveals significant interfacial differences. The sample annealed for 2 h exhibits a uniform coating interface composition without the large amorphous regions observed in Figure 9a. In contrast, the 1 h annealed sample shows incomplete reaction at the diamond–coating interface: due to insufficient annealing time, tungsten has not fully crystallized with carbon, resulting in an amorphous region. As illustrated in Figure 9b, this amorphous zone bonds to the coating through diamond/WC association. In the 2 h annealed sample, diamond graphitization inevitably occurs; however, extended annealing time promotes atomic rearrangement, yielding a smaller amorphous region bonded to the coating as diamond/W_2_C (Figure 9g). Figure 9c reveals distinct grain boundaries and defects between WC/W_2_C phases in the coating’s central region. Prolonged heat treatment transforms this central zone primarily into WC (Figure 9h), with Figure 9j demonstrating its ordered crystalline structure. Both Figure 9d,i show direct W_2_C bonding between coating and copper matrix without significant amorphous regions, though Figure 9i exhibits a sharper coating–matrix interface. These microstructural evolution trends correlate directly with composite thermal conductivity variations. Specifically, the 2 h annealed sample achieves peak thermal conductivity due to its minimized amorphous region, densified interface, and enhanced thermal transfer efficiency. Crucially, amorphous phase reduction and interfacial structural optimization constitute key determinants for thermal conductivity enhancement.

To study the interfacial heat transfer between different phases, commonly used models include AMM [47,48] and DMM [24]. The h for phonon-dominated interfaces (such as metal/diamond and carbide/diamond) is calculated using the Acoustic Mismatch Model (AMM), while for electron-controlled interfaces (like Cu/W), the Extended Diffusion Mismatch Model (DMM) is employed [29]. These two models are widely used in the study of diamond–copper composites to analyze interfacial thermal conductivity and help clarify the interfacial heat transfer mechanisms between materials. Therefore, the interfacial thermal conductivity (*hc*) between different materials was calculated using AMM Equation (6) and DMM Equation (7), with the calculation parameters listed in Table 3.

**Table 3:** Calculation parameters for AMM and DMM [12,22,24,29,47,48,49].

		AMM			DMM			
	*λ* W/(m·K)	ρ kg/m^3^	Cp J/kg·K	ν m/s	γ J/m^3^·K^2^	ν_F_ m/s	T K	Z
diamond	1717	3520	512	13,430				
W	173	1930	134	5200	136	5.00 × 10^5^	298	2.03 × 10^10^
WC	121	15,700	132	4706				
W_2_C	1.139	17,200	325	3554				
Cu	385	8960	386	2801	98	1.60 × 10^6^	298	4.67 × 10^10^
a-C		2000	710	2072

(6) $$R=\frac{2{\left(\rho_{m}v_{m}+\rho_{d}v_{d}\right)}^{2}}{C_{m}\cdot \rho_{m}^{2}\cdot v_{m}^{2}\cdot \rho_{d}\cdot v_{d}}{\left(\frac{v_{d}}{v_{m}}\right)}^{2}$$

(7) $$h_{c}=\frac{Z_{m}\cdot Z_{d}}{4(Z_{m}+Z_{d})}\;\mathrm{with}\;Z=C_{e}\cdot v_{F}$$

In these equations, $h_{c}$ denotes the interfacial thermal conductance and R denotes the interfacial thermal resistance. ρ, v, and C represent the density, Debye sound velocity, and specific heat capacity of the materials, respectively; the *m* and *d* subscripts indicate the matrix and reinforcement phase, respectively. $C_{e}$ and $v_{F}$ refer to the electronic heat capacity per unit volume and Fermi velocity of the electrons, respectively. $C_{e}=\gamma \cdot T$, where *γ* and *T* denote the electronic specific heat coefficient and temperature coefficient, respectively. Note that the interfacial thermal conductance and resistance are reciprocal and can be converted into one another.

**Figure 10:**
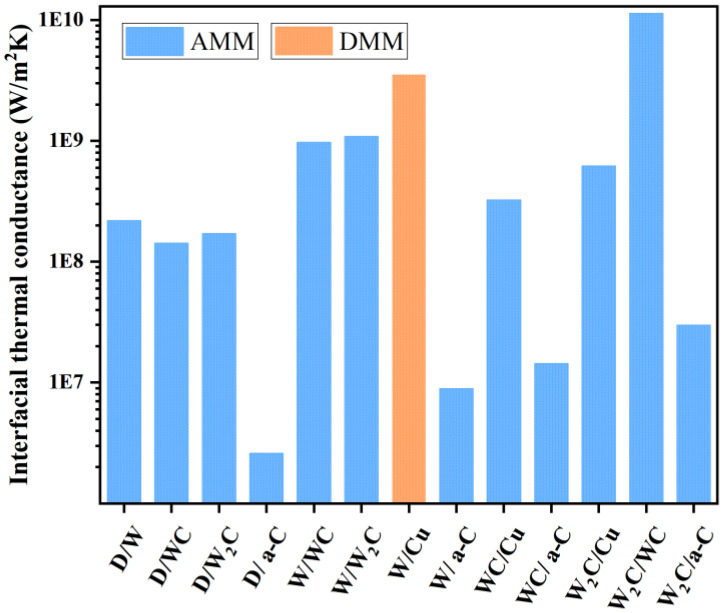
AMM- and extended DMM- derived interfacial thermal conductance values.

The calculation results are shown in Figure 10. The interface thermal conductivities between diamond and tungsten (W) as well as its carbides are all higher than that of amorphous carbon (a–C). However, as indicated in Figure 9, diamond inevitably forms a layer of amorphous carbon on its surface after annealing, which then combines with W and its carbides. The interface thermal conductivity between a-C and various materials is relatively low, with the lowest being between a–C and diamond (2.59 MW/m^2^K) and the highest between a–C and W_2_C (29.78 MW/m^2^K). In the coating, W and its carbides combine, achieving the highest interface thermal conductivity (11,400 MW/m^2^K) between W_2_C and WC due to better acoustic matching. The interface thermal conductivity between W and W_2_C is slightly higher than that of WC. Finally, at the junction between the coating and copper, W/Cu has a higher thermal conductivity than WC and W_2_C due to electronic conduction. However, at the peak thermal conductivity, the interface between copper and the coating is formed with W_2_C. Although the thermal conductivity of W_2_C is lower than that of WC and W, W_2_C can serve as an intermediate layer, helping to regulate the phonon vibration modes between adjacent materials, thereby reducing phonon scattering at the interface and increasing the thermal conductivity. The interface thermal conductivity of the combination with W_2_C is higher than that of the combination with WC, regardless of whether it is Cu as the matrix or diamond as the reinforcement. Furthermore, in actual interface reaction, the tungsten layer will first react with diamond to form W_2_C before forming WC, making it nearly impossible to have a diamond/W combination after the reaction. Therefore, the best achievable interface structure in practice is diamond/W_2_C/WC/W_2_C/Cu. The above analysis results have theoretical guiding value for the optimization of interface components of diamond–copper composite materials.

## 4. Conclusions

This study successfully prepared high thermal conductivity diamond–copper composites by surface modification of diamond and depositing tungsten coatings on diamond particles using magnetron sputtering, followed by annealing at 1100 °C for 2 h. Experimental results indicate that when the interface layer consists of 2 wt.% W, 92 wt.% WC, and 6 wt.% W_2_C, the thermal conductivity of the composite reaches 640 W/(m·K), which is the highest reported value for hot-pressed composites with diamond volume content not exceeding 50%. This demonstrates the significant enhancement in thermal conductivity due to the improved interface structure. Additionally, calculations of interface thermal conductivity using the acoustic mismatch model (AMM) and the diffusion mismatch model (DMM) further validate that diamond/W_2_C/WC/W_2_C/Cu is the best achievable interface structure arrangement. This optimization of the interface structure not only strengthens the bonding between diamond and copper but also effectively reduces thermal interface resistance, thereby improving overall thermal conductivity. These findings provide theoretical support and design guidance for interface engineering in diamond–copper composites, highlighting the potential for precise control of interfaces at the microstructural level to enhance thermal conductivity.

Future research will focus on further optimizing the preparation process, particularly exploring ways to increase the volume fraction of diamond and reduce the porosity of the material. With an increase in diamond content, the overall thermal conductivity of the material is expected to improve further. Moreover, by strictly controlling the composition and microstructure of the interface layer, it is hoped to reduce the generation of amorphous carbon and avoid the emergence of low-conductivity phases, thereby maximizing the thermal conductivity of diamond–copper composites. The results of this study provide a reference for the design and industrial application of high thermal conductivity diamond–copper composites and lay a foundation for the development of other efficient thermal management materials.

## Figures and Tables

**Table 1:** Bands in the Raman spectrum.

Peak Assignment	Wave Number (cm^−1^)	Substance	Hybridization of C Atoms
WC–O	261,323		
WC–O/WC	703	Diamond-like distorted graphite	Sp^2^ Sp^2^, Sp^3^
WC	805		
D	1384		
D/G	1556		
G	1589		

## Data Availability

The original contributions presented in this study are included in the article material. Further inquiries can be directed to the corresponding authors.
